# The effect of dextrose prolotherapy on pain and functional outcomes in chronic shoulder pain: A systematic review and meta-analysis of randomized controlled trials

**DOI:** 10.5339/qmj.2026.62

**Published:** 2026-09-17

**Authors:** Rizki Darmawan, Yose Waluyo, Nuralam Sam, Ahmad Taufik Fadillah Zainal, Moh Anfasa Giffari Makkaraka, Rivaldo Go

**Affiliations:** 1 Department of Physical Medicine and Rehabilitation, Faculty of Medicine, Hasanuddin University, Makassar, South Sulawesi, Indonesia; 2 Faculty of Medicine, Hasanuddin University, Makassar, South Sulawesi, Indonesia

**Keywords:** Dextrose prolotherapy, pain, functional outcome, chronic shoulder pain

## Abstract

**Background:**

Shoulder pain is a common musculoskeletal condition that substantially affects daily activities and work and imposes a considerable socioeconomic burden. Dextrose prolotherapy has been proposed as a regenerative intervention for musculoskeletal disorders, but previous reviews have primarily focused on specific shoulder conditions rather than chronic shoulder pain across different etiologies. This study evaluated the effects of dextrose prolotherapy on pain and functional outcomes in adults with chronic shoulder pain compared with control interventions.

**Methods:**

PubMed, ScienceDirect, and the Cochrane Library were searched on January 20, 2025. Three reviewers independently screened studies according to predefined eligibility criteria. Risk of bias was assessed using the Cochrane Risk of Bias 2 tool, and pooled effects were estimated using standardized mean differences (SMDs).

**Results:**

Among 585 identified records, 12 randomized controlled trials were included in the systematic review, and seven were eligible for each quantitative synthesis. The overall pooled effect on pain was not statistically significant (SMD −0.19; 95% confidence interval [CI], −0.84 to 0.46). The pooled effect on functional outcomes was also not statistically significant (SMD −0.72; 95% CI, −1.85 to 0.41). Exploratory subgroup analysis suggested lower pain scores after repeated injections (at least three sessions; SMD −0.80; 95% CI, −1.26 to −0.35), whereas a single injection was associated with higher pain scores than control (SMD 0.60; 95% CI, 0.04 to 1.16). These subgroup findings were accompanied by substantial heterogeneity and were not derived from direct head-to-head comparisons.

**Conclusion:**

Current evidence does not demonstrate a statistically significant overall benefit of dextrose prolotherapy for pain or functional outcomes in chronic shoulder pain. Repeated injections may be associated with greater pain reduction than a single injection, but this exploratory finding requires confirmation in adequately powered, diagnosis-specific randomized trials using standardized injection protocols.

## 1. INTRODUCTION

Shoulder pain is a common musculoskeletal complaint that can become chronic when it persists for more than three months, significantly affecting daily activities and socioeconomic well-being.^1–3^ It involves various structures, including the rotator cuff, joints, and surrounding tissues, with moderate pain levels reported in conditions such as rotator cuff pathology, labral tears, frozen shoulder, and bursitis.^4,5^ The incidence is high, ranging from 7.7 to 62 per 1000 people annually.^6^

Dextrose prolotherapy is an injection-based regenerative intervention in which a hypertonic dextrose solution is administered to painful tendons, ligaments, entheses, periarticular soft tissues, or intra-articular structures. Dextrose acts as a hyperosmolar proliferant rather than as a conventional analgesic drug and is intended to induce a controlled local inflammatory response that promotes growth-factor release, fibroblast activity, collagen synthesis, and extracellular-matrix remodeling. Concentrations between 12.5% and 25% are commonly used for proliferative applications, although lower concentrations may be used for perineural or superficial injection techniques, and biological effects may vary according to concentration and target tissue.^7,8^

Evidence suggests that dextrose prolotherapy may reduce pain and improve joint function in some musculoskeletal disorders, although its comparative effectiveness against interventions such as hyaluronic acid and platelet-rich plasma (PRP) remains uncertain.^9^ Proposed mechanisms include macrophage and fibroblast recruitment, increased expression of transforming growth factor beta 1 (TGF-beta1) and vascular endothelial growth factor (VEGF), neovascularization, and collagen remodeling.^10^ Reported adverse effects are usually transient and include post-injection pain, localized swelling, stiffness, and bruising; uncommon but potentially serious complications include infection, bleeding, allergic reactions, and neurovascular injury. Dextrose prolotherapy is not specifically approved by the United States Food and Drug Administration (FDA) for chronic shoulder pain and is therefore used off-label for this indication.

Most previous systematic reviews have focused on individual shoulder pathologies, limiting conclusions across the broader clinical spectrum of chronic shoulder pain. This review therefore evaluated the effects of dextrose prolotherapy on pain and functional outcomes across different chronic shoulder conditions and explored whether outcomes differed between single and repeated treatment schedules. Because these conditions and protocols are clinically heterogeneous, we considered frequency-based subgroup analyses exploratory.

## 2. MATERIALS AND METHODS 

### 2.1. Protocol and registration

This systematic review was registered in PROSPERO (CRD42023417709) and conducted according to PRISMA (Preferred Reporting Items for Systematic Reviews and Meta-Analyses) 2020 guidelines.

### 2.2. Literature searching

Three reviewers performed a literature search on January 20, 2025, using PubMed, ScienceDirect, and the Cochrane Library with keywords related to shoulder pain and dextrose injection (Table 1). The strategy was adapted for each database, and reference lists of included studies were manually screened to identify additional relevant studies.

### 2.3. Study selection

Studies were eligible if they were randomized controlled trials published within the preceding 15 years, enrolled adults with chronic shoulder pain lasting at least three months, compared dextrose prolotherapy with standard therapy, placebo, or another active comparator, and reported pain or functional outcomes. Studies published in English or Indonesian were considered. Records were imported into Rayyan for duplicate removal, after which three reviewers independently screened titles, abstracts, and full texts according to the predefined eligibility criteria.

### 2.4. Data collection

The following information was extracted from eligible randomized controlled trials: author and publication year, country, diagnosis, sample size, participant age, dextrose concentration and injection protocol, injection site, injection frequency, comparator, co-interventions, outcome measures, assessment time points, operator or injector specialty when reported, and principal findings. Requests for missing numerical data were limited to studies that met the final eligibility criteria after full-text assessment and were considered for quantitative synthesis. When possible, missing values were calculated from reported statistics; studies with insufficient quantitative data were retained in the qualitative synthesis but excluded from the relevant meta-analysis.

### 2.5. Risk of bias and study quality assessment

Risk of bias was assessed using the Cochrane Risk of Bias 2 tool across five domains: bias arising from the randomization process, including sequence generation and allocation concealment; bias due to deviations from intended interventions; bias due to missing outcome data; bias in outcome measurement; and bias in selection of the reported result. Each domain and the overall judgment were classified as low risk, some concerns, or high risk.

### 2.6. Statistical analysis

The predefined primary outcomes were pain intensity, measured using the Visual Analog Scale (VAS) or Numerical Rating Scale (NRS), and functional status, measured using the Shoulder Pain and Disability Index (SPADI) or Disabilities of the Arm, Shoulder and Hand (DASH) score. Range of motion (ROM), patient satisfaction, and imaging-based structural outcomes were treated as secondary outcomes and synthesized descriptively. Because different validated scales were used, pooled effects were expressed as standardized mean differences (SMDs) with 95% confidence intervals (CIs). For quantitative synthesis, the assessment closest to approximately 12 weeks was selected whenever available. Exploratory subgroup analyses compared single with repeated injections of at least three sessions. Heterogeneity was evaluated using the I^2^ statistic and Cochran chi-square test. A fixed-effect model was used when I^2^ was below 50%; otherwise, a DerSimonian-Laird random-effects model was applied. No multiplicity adjustment was applied because the subgroup analyses were exploratory rather than confirmatory.

### 2.7. Publication bias

Small-study effects and possible publication bias were explored visually using funnel plots. Because each meta-analysis included fewer than 10 studies, funnel-plot symmetry was considered descriptive only and was not interpreted as definitive evidence for or against publication bias.

## 3. RESULTS

### 3.1. Results of study screening

**Figure 1.**
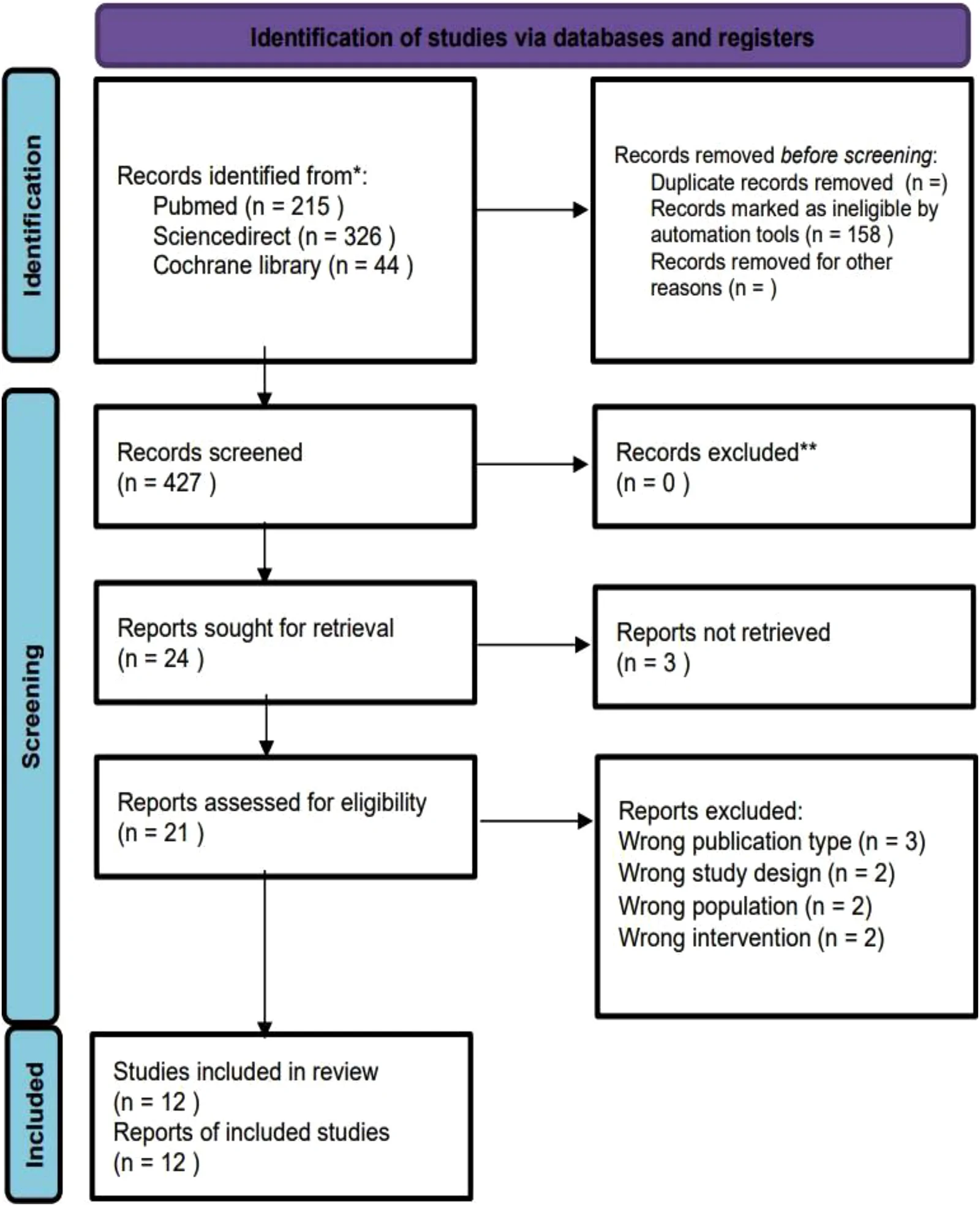
Results of study search and screening based on PRISMA guidelines.

A total of 585 studies were obtained from the study search (Figure 1). A total of 158 duplicate studies were excluded. The screening continued by reviewing the records (titles and abstracts) of the remaining 427 studies. Based on the desired study criteria, reviewers excluded 403 records due to irrelevant context, 3 studies with inaccessible reports, 3 studies with inappropriate publication types, 2 studies with inappropriate research designs, 2 studies with populations that did not meet the criteria, and 2 studies using inappropriate interventions. In the end, 12 inclusion studies were obtained for use in the quantitative synthesis in this study. The overall study population included 530 individuals with chronic shoulder pain disorders.

### 3.2. Studies’ risk of bias

**Figure 2.**
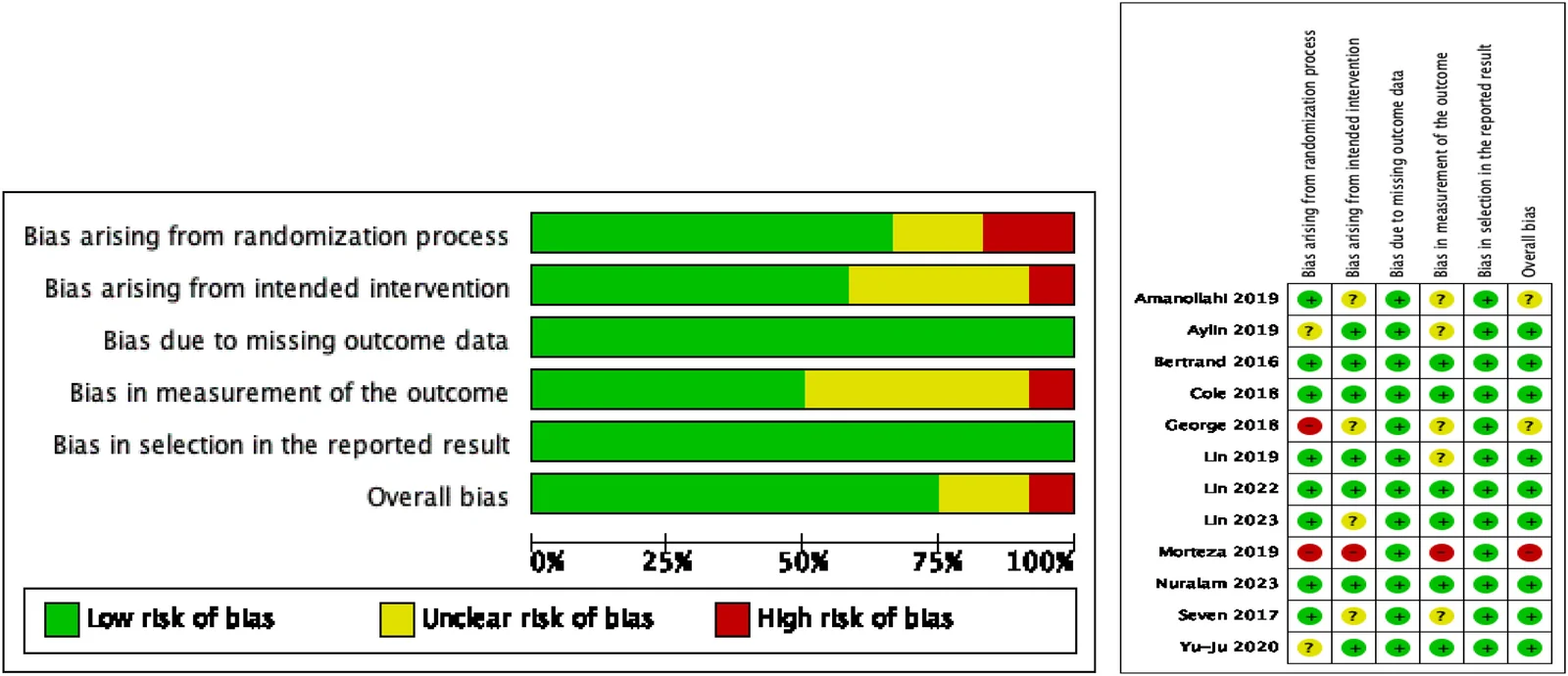
Risk-of-bias assessment of the included randomized controlled trials. Left: distribution of judgments for each assessed domain and overall bias across the included studies. Right: study-level judgments for each domain and overall bias. Green (+), low risk of bias; yellow (?), unclear risk of bias; red (−), high risk of bias.

Overall, the majority of risk-of-bias domains across the included studies were judged to have low risk. The outcome-measurement domain showed an approximately equal distribution between low-risk judgments and judgments of some concerns or high risk (Figure 2). One trial (Kazempour Mofrad et al.^11^ was judged to have high risk in several domains, whereas another trial by George et al.^12^) raised some concerns in multiple domains. Reporting of allocation concealment was incomplete in several trials and contributed to concerns within the randomization-process domain.

### 3.3. Characteristics of included studies

Twelve randomized controlled trials (RCTs), including 530 participants, evaluated dextrose prolotherapy for chronic shoulder pain. The characteristics of the included studies are summarized in Table 2. Sample sizes ranged from 9 to 101 participants, and mean ages ranged from 46 to 60 years. Comparators included saline placebo, corticosteroid injection, physiotherapy without injection, platelet-rich plasma, and lidocaine. Dextrose concentrations ranged from 5% to 25%, while injection schedules ranged from one to six sessions. Most trials used ultrasound guidance and targeted the supraspinatus tendon, subacromial bursa, entheses, glenohumeral or acromioclavicular structures, or periarticular pain points. The specialty and experience of the injector were not consistently reported, preventing analysis of operator-related effects.

The included trials enrolled clinically heterogeneous populations with rotator cuff tendinopathy or tears, supraspinatus tendinosis, subacromial bursitis, and adhesive capsulitis. Diagnoses were commonly supported by ultrasound or magnetic resonance imaging (MRI). Injection concentration, volume, target, frequency, comparator, follow-up duration, and permitted co-interventions also varied. Physiotherapy and medication use, including nonsteroidal anti-inflammatory drugs, were inconsistently reported across trials.

Primary outcomes were assessed using the VAS or NRS for pain, and the SPADI or DASH for function; lower scores indicated less pain or disability. Secondary outcomes included shoulder ROM, patient satisfaction, and ultrasonographic measures such as tendon thickness, tendon echogenicity, elasticity, and subacromial bursa thickness. These secondary outcomes were summarized qualitatively and were not included in the primary pooled analyses.

### 3.4. Effect of dextrose prolotherapy on pain

Of the 12 included RCTs, seven provided sufficiently comparable VAS or NRS data at the assessment closest to approximately 12 weeks and were included in the pain meta-analysis.^13–19^ Studies without compatible pain-scale data or an adequately aligned assessment time point were retained in the qualitative synthesis but excluded from this pooled analysis.^11,12,20–22^

**Figure 3.**
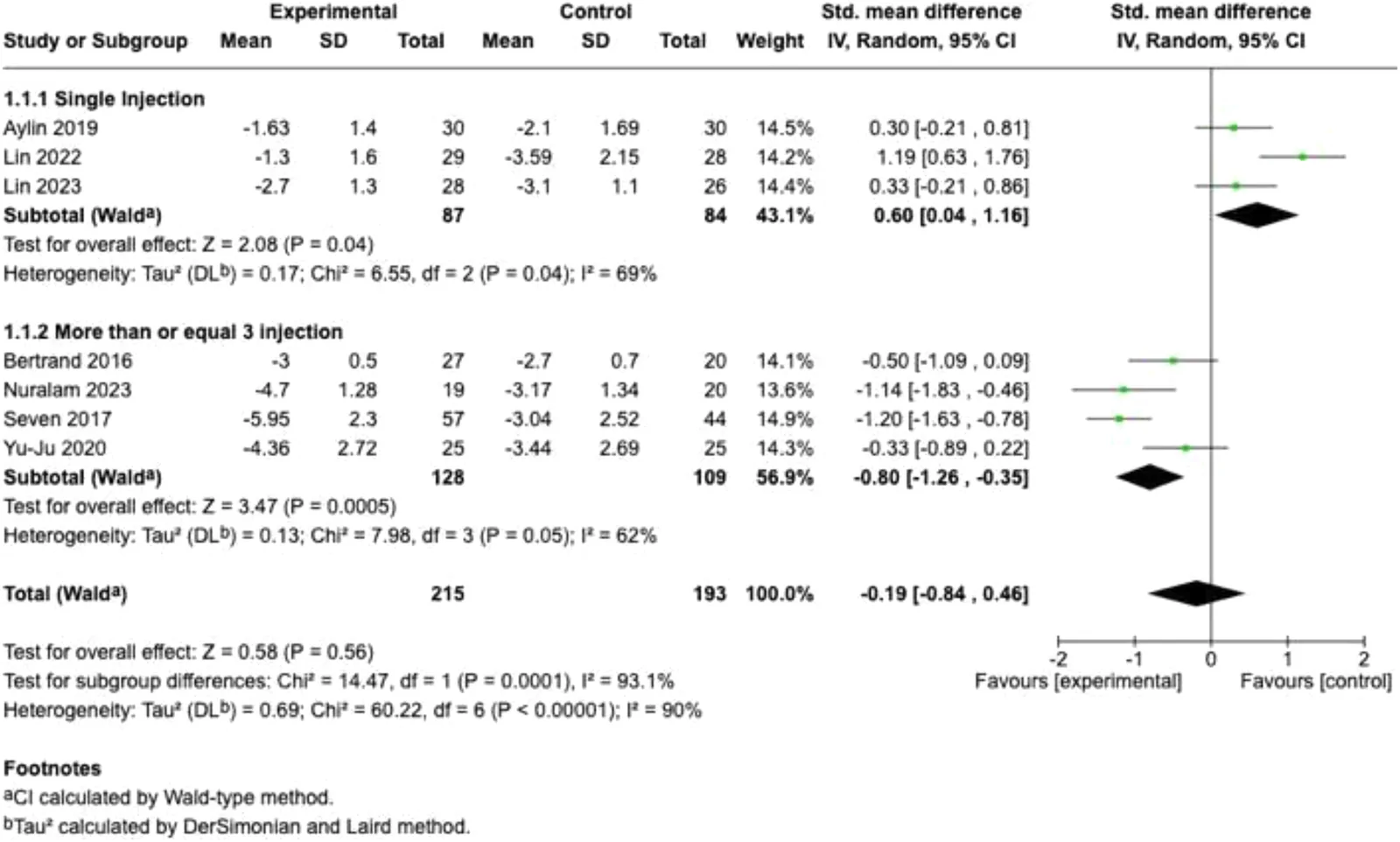
Forest plot of the effect of dextrose prolotherapy on pain, stratified by injection frequency. Subgroups comprise a single injection and repeated treatment with at least three injections. Effects are expressed as standardized mean differences (SMDs) with 95% confidence intervals (CIs), pooled using an inverse-variance random-effects model. Squares represent study estimates, with size proportional to study weight; horizontal lines indicate 95% CIs. Diamonds represent subgroup and overall pooled estimates. Negative SMDs favor dextrose prolotherapy. IV, Inverse variance; SD, Standard deviation.

Exploratory subgroup analysis categorized studies as single injection or repeated treatment comprising at least three injection sessions. For single injection, the pooled SMD was 0.60 (95% CI, 0.04 to 1.16; I^2^ = 69%; subgroup-effect *p* = 0.04), indicating higher pain scores in the dextrose group than in the control group. For repeated treatment, the pooled SMD was −0.80 (95% CI, −1.26 to -0.35; I^2^ = 62%; subgroup-effect *p* = 0.0005), indicating lower pain scores after dextrose prolotherapy. The overall pooled effect was not statistically significant (SMD −0.19; 95% CI, −0.84 to 0.46; I^2^ = 90%; *p* = 0.56). The test for subgroup differences was statistically significant; however, this indirect comparison was exploratory, was not adjusted for multiple testing, and cannot establish that repeated injections are superior to a single injection. The substantial overall heterogeneity indicates that the pooled estimate may not represent a single common treatment effect (Figure 3).

### 3.5. Effect of dextrose prolotherapy on functional outcomes

Seven RCTs reported sufficiently comparable SPADI or DASH data at the assessment closest to approximately 12 weeks and were included in the functional-outcome meta-analysis.^11,12,14,17–19,23^ Studies that did not use a compatible functional scale or reported only substantially earlier assessments were retained in the qualitative synthesis but excluded from this pooled analysis.^11,13,15,16,22^

For single injection, the pooled SMD for functional outcomes was -0.04 (95% CI, −1.03 to 0.94; I^2^ = 84%; *p* = 0.94). For repeated treatment comprising at least three sessions, the pooled SMD was −1.21 (95% CI, −3.09 to 0.67; I^2^ = 97%; *p* = 0.21). The overall effect was not statistically significant (SMD −0.72; 95% CI, −1.85 to 0.41; I^2^ = 96%; *p* = 0.21), and no statistically significant subgroup difference was identified. The very high heterogeneity indicates substantial variation in populations, diagnoses, intervention protocols, comparators, co-interventions, follow-up timing, and functional instruments. Accordingly, the functional findings should be interpreted as uncertain rather than as evidence of a consistent clinically meaningful benefit (Figure 3).

### 3.6. Assessment of small-study effects and publication bias

**Figure 4.**
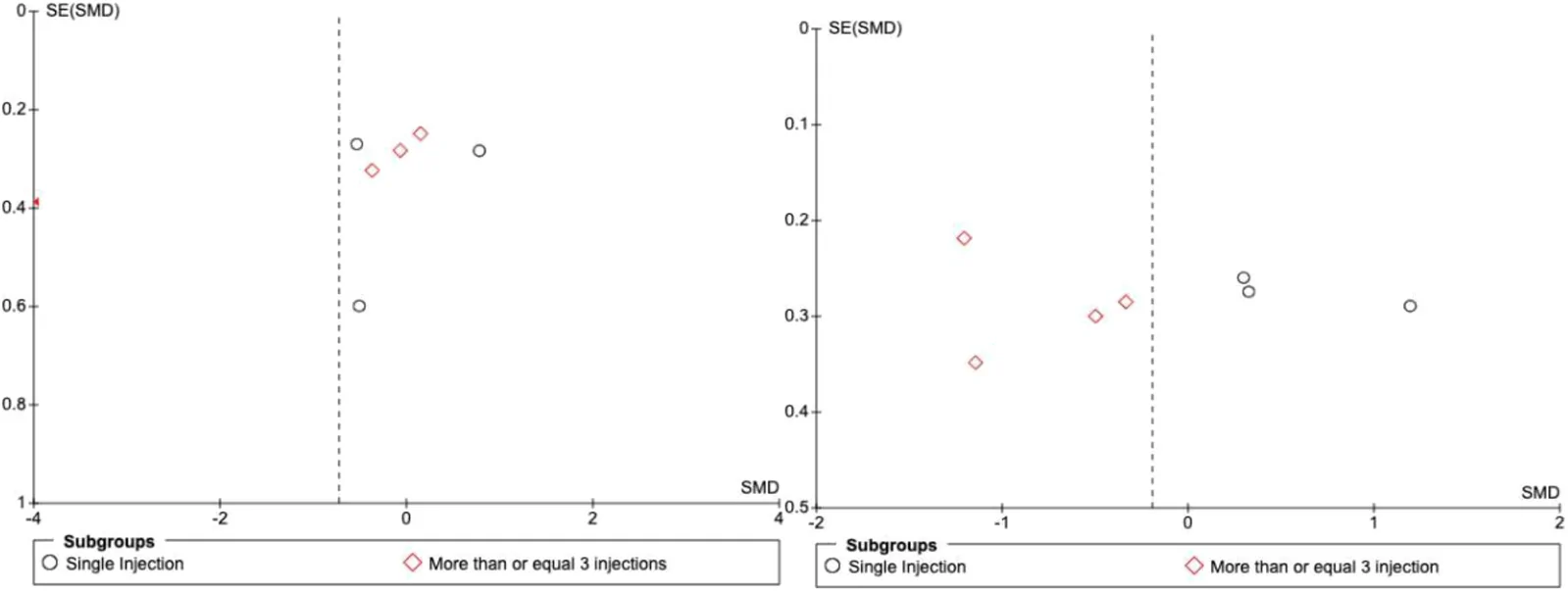
Funnel plots for pain and functional outcomes.

The pain-outcome funnel plot appeared asymmetric, with single-injection studies predominantly showing positive SMDs and repeated-injection studies predominantly showing negative SMDs. This distribution may reflect clinical and methodological differences between intervention protocols rather than publication bias. Because only seven studies were included, the plot had insufficient power for a reliable assessment of funnel-plot asymmetry and was interpreted descriptively (Figure 4).

The functional-outcome funnel plot appeared comparatively symmetric, although the small number of included studies and their moderate-to-small sample sizes precluded a definitive conclusion regarding publication bias or other small-study effects (Figure 4).

## 4. DISCUSSION

This systematic review found no statistically significant overall effect of dextrose prolotherapy on pain or functional outcomes in chronic shoulder pain. Exploratory subgroup analysis suggested lower pain scores after at least three injections and higher pain scores after a single injection compared with control interventions. This apparent frequency-related pattern is hypothesis-generating rather than confirmatory because it arose from indirect comparisons across different trials rather than from randomized head-to-head comparisons of treatment schedules. The high heterogeneity further limits inference regarding a single underlying treatment effect.

Dextrose prolotherapy was compared with saline, corticosteroids, platelet-rich plasma, lidocaine, and physiotherapy. This range of comparators broadens the clinical context of the review but also contributes to heterogeneity because comparator efficacy, duration of action, and accompanying rehabilitation differ. Most studies used ultrasound guidance, which may improve target localization and reduce variation from inaccurate needle placement; however, the specialty and experience of the injector were rarely reported. Consequently, operator-related effects could not be examined.

Conservative management of chronic shoulder pain commonly includes activity modification, analgesia, and diagnosis-specific physiotherapy, with injection therapy considered according to the underlying disorder and treatment response.^24^ Sabaah and Nassif also compared dextrose prolotherapy with platelet-rich plasma and corticosteroid injections in rotator cuff tendinopathy.^25^ A subsequent systematic review by Sony et al. focused on rotator cuff disorders, providing a narrower diagnostic context than the present review of chronic shoulder pain across different etiologies.^26^

The observed pain reduction after repeated injections is biologically plausible because serial exposure may provide repeated mechanical and hyperosmolar stimulation during tissue remodeling. Nevertheless, dextrose concentration ranged from 5% to 25%, and injection volume, anatomical target, and treatment interval differed substantially. These concentrations may not be biologically equivalent: lower concentrations may predominantly exert neuromodulatory or noninflammatory effects, whereas higher concentrations are intended to provoke a proliferative response. Concentration-specific subgroup analysis was not performed because too few trials were available within each concentration category to yield stable estimates.

Pain improvement did not consistently translate into statistically significant functional recovery. Function depends not only on pain intensity but also on joint stiffness, tendon integrity, muscle strength, ROM, rehabilitation adherence, disease chronicity, and diagnosis. Several trials reported ultrasonographic changes in tendon thickness, echogenicity, elasticity, or bursal thickness, providing objective information beyond symptom scales; however, the direction and clinical significance of these structural findings were inconsistent and could not be pooled. Therefore, the current evidence does not support recommending a specific dextrose regimen solely on the basis of the frequency subgroup analysis.

The proposed mechanism involves hyperosmolar cellular stress and local tissue irritation followed by inflammatory-cell recruitment, growth-factor signaling, fibroblast proliferation, collagen deposition, and extracellular-matrix remodeling.^27,28^ Additional hypotheses include neuromodulatory effects and improved local perfusion.^29,30^ Although these mechanisms may explain delayed or cumulative effects after repeated treatment, they remain incompletely established in human shoulder disorders and may differ according to dextrose concentration, tissue target, and underlying pathology.

This review has several limitations. Statistical heterogeneity was substantial for pain (I^2^ = 90%) and functional outcomes (I^2^ = 96%), reflecting variation in diagnoses, disease mechanisms, dextrose concentrations, injection volumes and sites, treatment frequency, comparators, co-interventions, outcome instruments, and follow-up timing. Pooling adhesive capsulitis, bursitis, rotator cuff tendinopathy, and tendon tears was consistent with the review’s broad chronic-shoulder-pain objective but may obscure diagnosis-specific effects. Diagnosis-specific and concentration-specific subgroup analyses were not feasible because too few trials were available in each category. Outcomes were selected at the time point closest to approximately 12 weeks, but residual temporal misalignment remained. Physiotherapy, medication use, and other co-interventions were inconsistently reported and may have confounded treatment effects. Allocation concealment was not clearly described in several trials. SMDs were necessary to combine different validated scales but are less intuitive clinically than scale-specific mean differences. Moreover, subgroup testing was exploratory and unadjusted for multiplicity, and funnel plots based on fewer than 10 studies cannot reliably exclude publication bias. Larger, adequately concealed, diagnosis-specific RCTs should directly compare single and repeated schedules, standardize dextrose concentration and injection technique, control co-interventions, and report clinically interpretable outcomes at harmonized follow-up intervals.

## 5. CONCLUSION

The available randomized evidence does not demonstrate a statistically significant overall benefit of dextrose prolotherapy for pain or functional outcomes in chronic shoulder pain. Repeated treatment comprising at least three injections was associated with lower pain scores in an exploratory subgroup analysis, whereas a single injection was not beneficial; however, this finding was based on indirect between-study comparisons and should not be interpreted as proof of superiority. Functional improvement was not statistically significant, and substantial clinical and statistical heterogeneity reduced certainty in all pooled estimates. Dextrose prolotherapy may remain an option in selected patients after individualized clinical assessment, but definitive recommendations regarding diagnosis, concentration, injection site, and treatment frequency require larger, standardized, head-to-head randomized trials.

## ACKNOWLEDGEMENT

The contributions of all six authors to this work are gratefully acknowledged. Their individual roles are detailed in the Authors’ Contribution statement.

## AUTHORS’ CONTRIBUTIONS

RD: Conceptualization, supervision, methodology, investigation, project administration, writing-original draft, review & editing. YW: Methodology, validation, formal analysis, writing-original draft. NS: Software, validation, data curation, resources. ATFZ: Investigation, data curation, writing-original draft. MAGM: Data curation, software, writing- review & editing. RG: Data curation.

## CONFLICTS OF INTEREST

The author(s) declare that there is no conflict of interest.

## DISCLOSURE OF AI USE

The authors declare that no artificial intelligence (AI) tools were used in the preparation of this manuscript.

## DATA AVAILABILITY

The author(s) declare that no data were generated or analyzed for this study.

## FUNDING

The author(s) received no financial support for the research, authorship, and/or publication of this article.

## ETHICAL APPROVAL

Not applicable. This study is a systematic review and meta-analysis of previously published data and did not involve recruitment of participants or collection of new individual-level data.

## Figures and Tables

**Table 1.** Search strategy for each database.

Database	Search Strategy
Pubmed	("Shoulder Pain" [Mesh] OR "shoulder pain" OR "chronic shoulder pain" OR "rotator cuff syndrome" OR "rotator cuff tendinopathy" OR "rotator cuff tear" OR "subacromial bursitis" OR "adhesive capsulitis" OR "frozen shoulder" OR "glenohumeral osteoarthritis" OR "shoulder osteoarthritis") AND ("Dextrose"[Mesh] OR "dextrose injection" OR "prolotherapy" OR "proliferation therapy" OR "perineural injection treatment" OR "perineural injection therapy")
Science Direct	TITLE-ABSTR-KEY ("shoulder pain" OR "rotator cuff tendinopathy" OR "rotator cuff tear" OR "adhesive capsulitis" OR "frozen shoulder")AND ("dextrose injection" OR prolotherapy OR "perineural injection")
Cochrane Library	("shoulder pain" OR "chronic shoulder pain" OR "rotator cuff syndrome" OR "rotator cuff tendinopathy" OR "rotator cuff tear" OR "subacromial bursitis" OR "adhesive capsulitis" OR "frozen shoulder" OR "glenohumeral osteoarthritis" OR "shoulder osteoarthritis") AND ("dextrose injection" OR "prolotherapy" OR "proliferation therapy" OR "perineural injection treatment" OR "perineural injection therapy")

**Table 2.** Characteristics of included studies.

No	Authors	Country	Diagnosis	Sample Size	Age (Years)	Intervention	Injection Site	Injection Frequency	Control Group	Outcome Measures	Time of Assessments	Main results
1	Chang et al.^19^	Taiwan	Rotator Cuff Disease and Bursitis confirmed with ultrasound	Intervention Group: 25 Control Group: 25	Intervention Group: 46.40 ± 9.59 Control Group: 47.72 ± 11.79	15% Dextrose	Bursal injections under ultrasound guidance	3 injections (every 2 weeks)	Normal saline	VAS SPADI AROM Flexion and Abduction Shoulder Thickness of the subacromial bursa Elasticity of supraspinatus tendon	0, 1, 4, and 12 weeks	VAS (at baseline and 12 weeks) Intervention: 7.36 ± 2.06 and 3.00 ± 2.45 (improved -4.36 ± 2.72)Control: 7.68 ± 1.70 and 4.24 ± 3.02 (improved -3.44 ± 2.69)AROM flexion in degrees (at baseline and 12 weeks) Intervention: 146.8 ± 23.04 and 168.8 ± 11.8 (improved 22.00 ± 21.89)Control 144.6±25.66 and 160.2 ± 22.8 (improved 15.60 ± 23.42)AROM abduction in degrees (at baseline and 12 weeks) Intervention: 117.4 ± 28.0 and 153.0 ± 29.5 improved 35.61 ± 23.02)Control: 115.6 ± 27.2 and 144.0 ± 33.3 (improved 28.4 ± 33.09)SPADI (at baseline and 12 weeks) Intervention: 50.16±27.31 and 19.14±20.51 (improved -31.00±25.83)Control: 57.80±26.96 and 28.64±28.02 (improved -29.16±29.12)Bursa thickness in mm (at baseline and 12 weeks) Intervention: 3.16±10.73 and 2.19±10.51 (improved -0.96±10.76)Control: 2.82±10.56 and 2.39±10.60 (improved - 0.43±10.58)Elasticity in m/s (at baseline and 12 weeks) Intervention: 60.59 ± 17.59 and 89.52 ± 24.56 (improved 28.93 23.79)Control: 56.83 ± 27.65 and 69.00 ± 22.41 (improved 12.17 ± 15.99)
2	Bertrand et al.^16^	Canada	Rotator Cuff Tendinopathy with ultrasound-confirmed supraspinatus tendinosis/tear	Intervention Group: 27 Control Group : Enth-Saline: 20 Superfic-Saline: 26	Intervention Group: 53.8±13.5 Control Group : Enth-Saline: 51.1±9.2 Superfic-Saline: 49.0±11.9	25% Dextrose	Supraspinatus, infraspinatus, teres minor, and other entheses around the shoulder joint	3 injections (0, 1, and 2 months)	Normal saline	Visual Analog Scale (VAS) Ultrasound Pathology Rating Scale (USPRS)	0, 3, and 9 months.	VAS (at baseline and 3 months) Intervention: 7.3 ± 0.4 and 4.3 ± 0.5 (improved 3.0 ± 0.5) Control: Enth-Saline: 6.9 ± 0.5 and 4.2 ± 0.7 (improved 2.7 ± 0.7) Superfic-Saline: 6.9 ± 0.4 and 4.2 ± 0.6 (improved 2.7 ± 0.6)VAS (at baseline and 9 months) Intervention: 7.3 ± 0.4 and 4.4 ± 0.5 (improved 2.9 ± 0.6) Control: Enth-Saline: 6.9 ± 0.5 and 5.1 ± 0.7 (improved 1.8 ± 0.7) Superfic-Saline: 6.9 ± 0.4 and 5.6 ± 0.6 (improved 1.3 ± 0.6)USPRS (baseline and improvement at 9 months) Intervention: 4.0 ± 0.4 and improved -0.3 ± 0.5 Control: Enth-Saline: 4.3 ± 0.5 and improved -0.6 ± 0.5 Superfic-Saline: 4.3 ± 0.4 and improved -0.6 ± 0.4
3	Kazempour Mofrad et al.^11^	Iran	Chronic Rotator Cuff Tendinopathy confirmed by magnetic resonance imaging (MRI).	Intervention Group: 32 Control Group: 33	Intervention Group: 52.5 ± 13.9 Control Group: 56.9 ± 13.6	12,5% Dextrose	Superficial injections around the shoulder joint and tender points along the suprascapular nerve	2 injections, 1-week apart	Physiotherapy without any injections	Shoulder Pain and Disability Index (SPADI)	0, 2 and 12 weeks	SPADI Pain Percentage (Baseline, 2 weeks, and 12 weeks) Intervention Group: 82.7 ± 6.5, 51.1 ± 23.3, and 47.2 ± 23.3 (improved -35.5) Control Group: 63.4 ± 9.6, 32.1 ± 11.7, and 30.5 ± 9.6 (improved -32.9)Disability Percentage (Baseline, 2 weeks, and 12 weeks) Intervention Group : 75.3 ± 12.2, 44.9 ± 19.1, and 39.5 ± 19.1 (improved -35.8) Control Group: 62.0 ± 5.5, 26.2 ± 8.4, and 22.6 ± 7.1 (improved -35.8)Total SPADI Score Percentage (Baseline, 2 weeks, and 12 weeks) Intervention Group: 78.1 ± 9.0, 47.3 ± 19.8, and 43.3 ± 19.8 ( improved -34.8 ) Control Group: 62.6 ± 5.8, 28.5 ± 8.2, and 26.9 ± 6.4 (improved -35.7)
4	Sam et al.^17^	Indonesia	Frozen Shoulder (Adhesive Capsulitis)	Intervention Group: 19 Control Group: 20	Intervention Group: 58.16 ± 6.34 Control Group: 57.60 ± 10.70	12,5% Dextrose	Glenohumeral joint, subacromial bursa, rotator cuff muscles (supraspinatus, infraspinatus, teres minor, subscapularis), acromioclavicular joint, and long head of biceps tendon	Four injections (0, 2, 4, and 6 weeks)	Normal saline	Ratio of MMP-1/TIMP-1 NRS (Numerical Rating Scale) DASH (Disability of the Arm, Shoulder, and Hand) score Range of Motion (ROM)	0, 6 and 12 weeks	Numerical Rating Scale (Baseline and Week 12) Intervention Group: 5.32 ± 1.003 and 0.62 ± 0.80 (improved -4.70) Control Group: 5.60 ± 0.68 and 2.43 ± 1.16 (improved -3.17)DASH Score (Baseline and Week 12) Intervention Group: 52.50 ± 13.69 and 10.01 ± 10.06 (improved -42.49) Control Group: 49.90 ± 9.67 and 13.33 ± 10.77 (improved -36.57)ROM (Baseline and Week 12) Flexion Intervention Group: 129.60 ± 16.10 and 151.05 ± 29.70 (improved +21.45) Control Group: 123.88 ± 19.64 and 140.75 ± 31.47 (improved +16.87)Internal Rotation Intervention Group: 61.05 ± 23.79 and 75.00 ± 12.91 (improved +13.95) Control Group: 53.13 ± 25.34 and 71.25 ± 14.13 (improved +18.12)External Rotation Intervention Group: 43.68 ± 20.00 and 66.58 ± 21.67 (improved +22.90) Control Group: 46.75 ± 26.03 and 55.00 ± 22.77 (improved +8.25)Ratio of MMP-1/TIMP-1 (Baseline and Week 12) Intervention Group: 0.0218 ± 0.015 and 0.0315 ± 0.009 (improved +0.0097) Control Group: 0.0187 ± 0.0145 and 0.0201 ± 0.0136 (improved +0.0014)
5	Sari and Eroglu^13^	Turkey	Rotator Cuff Lesions	Intervention Group: 30 Control Group: Platelet-Rich Plasma: 30 Corticosteroid: 30 Lidocaine: 30	Intervention and Control Group : 52.11 ± 10.78	20% Dextrose	Subacromial bursa (ultrasound-guided lateral subacromial injection)	Single injection	Platelet-Rich Plasma Corticosteroids Lidocaine	Visual Analog Scale (VAS) American Shoulder and Elbow Surgeons Standardized Shoulder Assessment Form (ASES) Western Ontario Rotator Cuff Index (WORC)	0, 3, 12, and 24 weeks	VAS (Baseline and 24 weeks)Intervention: 5.90 ± 0.88 and 3.10 ± 1.52 (Improved -2.80)Control: Platelet-Rich Plasma: 5.63 ± 1.00 and 2.57 ± 1.19 (Improved -3.06)Corticosteroids: 5.63 ± 0.93 and 3.77 ± 1.41 (Improved -1.86)Lidocaine: 5.47 ± 0.86 and 3.20 ± 1.19 (Improved -2.27)ASES (Baseline and 24 weeks)Intervention : 45.00 ± 9.42 and 60.37 ± 11.40 (Improved +15.37)Control: Platelet-Rich Plasma: 46.28 ± 8.61 and 63.87 ± 11.96 (Improved +17.59)Corticosteroids: 40.13 ± 8.18 and 55.63 ± 11.00 (Improved +15.50)Lidocaine: 47.27 ± 7.44 and 60.27 ± 11.92 (Improved +13.00)WORC (Baseline and 24 weeks)Intervention: 53.67 ± 8.43 and 91.27 ± 21.79 (Improved +37.60)Control: Platelet-Rich Plasma: 50.79 ± 6.48 and 79.46 ± 24.09 (Improved +28.67)Corticosteroids: 51.40 ± 7.73 and 93.90 ± 17.94 (Improved +42.50)Lidocaine: 52.13 ± 7.92 and 96.55 ± 20.43 (Improved +44.42)
6	Lin et al.^14^	Taiwan	Chronic supraspinatus tendinopathy	Intervention Group: 16 Control Group: 15	Intervention Group: 46.25 ± 5.69 Control Group: 48.60 ± 5.95	20% Dextrose	Supraspinatus tendon insertion site under ultrasound guidance	Single injection	Normal saline	VAS SPADI Shoulder AROM (flexion, rotation, abduction) Supraspinatus tendon ROI histogram via ultrasound.	0, 2 and 6 weeks	VAS (Baseline and 6 weeks)Intervention Group: 5.56 ± 0.81 and 5.13 ± 0.72 (improved -0.43)Control Group: 5.33 ± 0.82 and 4.87 ± 0.64 (improved -0.46)SPADI (Baseline and 6 weeks)Intervention Group: 60.50 ± 7.87 and 61.56 ± 4.68 (improved +1.06)Control Group: 65.00 ± 2.78 and 60.00 ± 4.90 (improved -5.00)Shoulder AROM (Baseline and 6 weeks) FlexionIntervention Group: 157.15 ± 13.40 and 159.38 ± 7.50 (improved +2.23)Control Group: 156.21 ± 6.51 and 161.20 ± 6.03 (improved +4.99)AbductionIntervention Group: 146.66 ± 13.96 and 148.69 ± 8.89 (improved +2.03) Control Group: 140.46 ± 13.35 and 145.93 ± 14.70 (improved +5.47)Internal RotationIntervention Group: 45.00 ± 8.17 and 45.00 ± 8.17 (improved 0.00)Control Group: 44.67 ± 7.32 and 44.67 ± 7.24 (improved 0.00)External Rotation Intervention Group: 57.50 ± 10.65 and 61.25 ± 8.27 (improved +3.75)Control Group: 60.00 ± 8.45 and 62.33 ± 4.17 (improved +2.33)Supraspinatus Tendon ThicknessIntervention Group: 6.93 ± 1.18 mm and 7.43 ± 1.18 mm (improved +0.50 mm)Control Group: 7.01 ± 0.58 mm and 7.16 ± 0.51 mm (improved +0.15 mm)
7	George et al.^12^	Malaysia	Focal supraspinatus tendinosis confirmed by ultrasound	Intervention Group: 7 Control Group: 5	Intervention Group: 60 Control Group: 58	12.5% Dextrose	Intratendinous injection into the focal area of tendinosis in the supraspinatus tendon under ultrasound guidance	Single injection	Standard physiotherapy without injection	Disability of Arm, Shoulder, and Hand (DASH) Pain score (subset of DASH). Sleep score. Range of motion (ROM) for shoulder abduction. Tendon echogenicity via ultrasound.	0 and 12 weeks	DASH (Baseline and 12 weeks)Intervention Group: 60.14 and 43.89 (improved -27.0%)Control Group: 56.86 and 46.68 (improved -17.9%)Pain score (Baseline and 12 weeks)Intervention Group: 3.29 and 1.86 (improved -43.5%)Control Group: 3.20 and 2.40 (improved -25.0%)Sleep score (Baseline and 12 weeks)Intervention Group: 3.29 and 2.15 (improved -34.6%)Control Group: 2.20 and 2.60 (worsened +18.2%)Range of motion (ROM) for shoulder abductionIntervention Group: Increase +20.0° Control Group: Decrease -12.0°Tendon echogenicity via ultrasound: Improved
8	Cole et al.^20^	Australia	Supraspinatus tendinopathy confirmed by ultrasound	Intervention Group: 17 Control Group: 19	Intervention Group: 51 ± 16 Control Group: 46 ± 15	25% Dextrose	Intervention group : Hypoechoic/anechoic areas of the supraspinatus tendon. Control group : Subacromial bursa adjacent to the supraspinatus tendon.	Single injection	Corticosteroid	Pain during overhead activities (five-point Likert scale: none, mild, moderate, severe, very severe). Shoulder satisfaction. Frequency of pain with activity or during sleep. Shoulder range of motion (ROM). Tendon changes on ultrasound.	0, 6, 12 and 24 weeks	Pain during overhead activities (Likert Scale: None to Very Severe) (Baseline and 24 weeks)Intervention Group: Moderate-Severe and Mild (Improved by 2 levels)Control Group: Moderate-Severe and Mild (Improved by 2 levels)Shoulder satisfaction (5-Point Scale: Very Dissatisfied to Very Satisfied) (Baseline and 24 weeks)Intervention Group: Dissatisfied and Very Satisfied (+3 levels)Control Group: Dissatisfied and Satisfied (+2 levels)Frequency of pain with activity or during sleep. (Baseline and 24 weeks)Intervention Group: Frequent and Rarely (-3 levels)Control Group: Frequent and Occasionally (-2 levels)Shoulder range of motion (ROM). (Baseline and 24 weeks) Forward flexionIntervention Group: 135° and 150° (improved +15°)Control Group: 140° and 150° (improved +10°)AbductionIntervention Group: 130° and 145° (improved +15°)Control Group: 135° and 145° (improved +10°)External rotationIntervention Group: 60° and 75° (improved +15°)Control Group: 65° and 75° (improved +10°)Tendon changes on ultrasound (Baseline and 24 weeks)Intervention Group: Present and Absent (complete healing)Control Group: Present and Reduced (partial healing)
9	Amanollahi et al.^22^	Iran	Rotator cuff tendinopathy confirmed by MRI.	Intervention Group: 28 Control Group: 29	Intervention Group: 59.4 ± 9.2 Control Group: 56.3 ± 8.3	5% Dextrose	Intervention Group: Subcutaneous injection to tender points around the shoulder, including anterior, posterior, lateral sides, and specific tendons (e.g., supraspinatus) Control Group : Subacromial bursa	Intervention Group: Three weekly injectionsControl Group: Single injection	Corticosteroid	VAS Shoulder ROM	0 and 4 weeks	VAS (Baseline and 4 weeks)Intervention Group : 6.5 ± 1.1 and 3.0 ± 1.2 (improved -3.5 ± 1.2)Control Group: 6.7 ± 0.9 and 4.2 ± 1.3 (improved -2.5 ± 1.4)Range of Motion (Baseline and 4 weeks)FlexionIntervention Group: 126.4 ± 14.0 and 150.4 ± 15.3 (improved +24.0°)Control Group : 122.0 ± 10.5 and 155.7 ± 12.8 (improved +33.7°)AbductionIntervention Group: 107.2 ± 11.8 and 128.2 ± 13.5 (improved +21.0°)Control Group: 103.5 ± 9.4 and 153.5 ± 10.7 (improved +50.0°)External RotationIntervention Group: 45.1 ± 7.5 and 55.1 ± 8.0 (improved +10.0°)Control Group: 43.2 ± 6.7 and 57.8 ± 7.4 (improved +14.6°)
10	Lin et al.^15^	Taiwan	Chronic subacromial bursitis	Intervention Group: 28 Control Group: 26	Intervention Group: 53.21 ± 9.15 Control Group: 57.46 ± 11.49	20% Dextrose	Subacromial bursa (anterior approach with ultrasound guidance)	Single injection	Corticosteroid	VAS SPADI Shoulder ROM Subacromial Bursa Thickness	0, 2, 6 and 12 weeks	VAS (Baseline and 12 weeks)Intervention Group: 7.3 ± 1.2 and 4.6 ± 1.3 (improved -2.7 ± 1.3)Control Group: 7.5 ± 1.1 and 4.4 ± 1.1 (improved -3.1 ± 1.1)SPADI (Baseline and 12 weeks)Intervention Group: 54.8 ± 9.5 and 36.9 ± 10.5 (improved -17.9 ± 10.5)Control Group: 55.2 ± 9.3 and 29.4 ± 9.3 (improved -25.8 ± 9.3)Shoulder ROM (Baseline and 12 weeks) FlexionIntervention Group: 125.6 ± 11.3 and 137.0 ± 11.0 (improved +11.4 ± 11.0)Control Group: 124.8 ± 12.0 and 144.3 ± 11.8 (improved +19.5 ± 11.8)AbductionIntervention Group: 102.3 ± 10.9 and 117.8 ± 11.0 (improved +15.5 ± 11.0)Control Group : 103.8 ± 11.5 and 126.3 ± 12.0 (improved +22.5 ± 12.0)Internal RotationIntervention Group: 54.5 ± 9.8 and 60.1 ± 9.7 (improved +5.6 ± 9.7)Control Group: 55.0 ± 10.0 and 65.8 ± 9.7 (improved +10.8 ± 9.7)External RotationIntervention Group: 42.5 ± 7.9 and 45.4 ± 7.7 (improved +2.9 ± 7.7)Control Group: 43.2 ± 8.0 and 51.0 ± 8.0 (improved +7.8 ± 8.0)Subacromial bursa thickness via ultrasound (Baseline and 12 weeks)Intervention Group: 3.8 ± 0.5 and 3.2 ± 0.6 (improved -0.6 mm)Control Group: 4.0 ± 0.6 and 2.8 ± 0.6 (improved -1.2 mm)
11	Seven et al.^18^	Turkey	Chronic rotator cuff lesions confirmed by MRI	Intervention Group: 57 Control Group: 44	Intervention Group: 50.19 ± 12.13 Control Group: 46.31 ± 10.6	25% Dextrose	Subacromial bursa and multiple sites around the rotator cuff (e.g., supraspinatus, infraspinatus, teres minor insertions)	Up to six sessions, with at least three injections applied	Standard physiotherapy	VAS SPADI WORC Shoulder ROM Patient satisfaction	O, 3, 6, 12 weeks and 1 year	VAS (Baseline, 12 weeks and 1 year)Intervention Group: 7.85 ± 1.29, 1.90 ± 1.80 and 0.89 ± 1.64 (improved -6.96 ± 1.64)Control Group: 7.36 ± 1.38, 4.32 ± 1.63 and 3.77 ± 2.15 (improved -3.59 ± 2.15)SPADI (Baseline, 12 weeks and 1 year)Intervention Group: 74.76 ± 18.54, 12.65 ± 11.76 and 7.66 ± 10.64 (improved -67.10 ± 10.64)Control Group: 68.62 ± 20.40, 40.23 ± 19.40 and 34.94 ± 19.14 (improved -33.68 ± 19.14)WORC (Baseline, 12 weeks and 1 year)Intervention Group: 32.21 ± 17.49, 84.33 ± 12.45 and 90.37 ± 10.12 (improved +58.16 ± 10.12)Control Group: 37.77 ± 16.03, 61.98 ± 15.98 and 69.08 ± 16.70 (improved +31.31 ± 16.70)Shoulder ROM (Baseline, 12 weeks and 1 year) FlexionIntervention Group: 125.8 ± 11.3, 160.3 ± 11.1 and 165.6 ± 11.5 (improved +39.8 ± 11.5)Control Group: 128.4 ± 10.8, 147.5 ± 11.0 and 150.0 ± 11.6 (improved +21.6 ± 11.6)AbductionIntervention Group: 108.6 ± 10.5, 147.2 ± 12.1 and 153.6 ± 11.9 (improved +45.0 ± 11.9)Control Group: 110.4 ± 11.2, 134.0 ± 11.0 and 137.8 ± 11.5 (improved +27.4 ± 11.5)
12	Lin et al.^30^	Taiwan	Chronic supraspinatus tendinosis confirmed by ultrasound	Intervention Group: 29 Control Group: 28	Intervention Group: 49.10 ± 8.44 Control Group: 52.18 ± 9.83	20% Dextrose	Supraspinatus tendon insertion	Single injection	Normal saline	VAS SPADI Shoulder ROM Supraspinatus tendon thickness	0, 2, 6 and 12 weeks	VAS (Baseline and 12 weeks) Intervention Group: 6.7 ± 1.4 and 5.4 ± 1.6 (improved -1.3 ± 1.6) Control Group: 6.8 ± 1.5 and 6.2 ± 1.5 (improved -3.59 ± 2.15) SPADI Intervention Group: 58.3 ± 8.9 and 51.0 ± 9.4 (improved -7.3 ± 9.4) Control Group: 59.1 ± 9.0 and 56.8 ± 9.1 (improved -2.3 ± 9.1) Shoulder ROM Flexion Intervention Group: 135.4 ± 11.8 and 140.6 ± 12.0 (improved +5.2 ± 12.0) Control Group: 134.9 ± 11.6 and 136.8 ± 11.8 (improved +1.9 ± 11.8) Abduction Intervention Group: 108.2 ± 10.3 and 110.7 ± 10.4 (improved +2.5 ± 10.4) Control Group: 107.9 ± 10.1 and 109.2 ± 10.2 (improved +1.3 ± 10.2) Internal Rotation Intervention Group: 60.4 ± 8.7 and 61.2 ± 8.7 (improved +0.8 ± 8.7) Control Group: 60.7 ± 8.5 and 61.1 ± 8.5 (improved +0.4 ± 8.5) External Rotation Intervention Group: 48.5 ± 7.4 and 49.2 ± 7.4 (improved +0.7 ± 7.4) Control Group: 48.6 ± 7.3 and 49.0 ± 7.3 (improved +0.4 ± 7.3) Supraspinatus tendon thickness Intervention Group: 4.5 ± 0.6 and 5.1 ± 0.6 (improved +0.6 mm) Control Group: 4.4 ± 0.5 and 4.4 ± 0.5 (improved by 0 mm)
